# In Vitro Evaluation of Different Dietary Methane Mitigation Strategies

**DOI:** 10.3390/ani9121120

**Published:** 2019-12-11

**Authors:** Juana C. Chagas, Mohammad Ramin, Sophie J. Krizsan

**Affiliations:** Department of Agricultural Research for Northern Sweden, Swedish University of Agricultural Sciences (SLU), Skogsmarksgränd, 90183 Umeå, Sweden; juana.chagas@slu.se (J.C.C.); mohammad.ramin@slu.se (M.R.)

**Keywords:** antimethanogenic, chemical inhibition, global warming, halogenated compound, macroalgae, methane production, methanogenic inhibitor, plant inhibitory compound

## Abstract

**Simple Summary:**

Dietary methane mitigation strategies do not necessarily make food production from ruminants more energy-efficient, but reducing methane (CH_4_) in the atmosphere immediately slows down global warming, helping to keep it within 2 °C above the pre-industrial baseline. There is no single most efficient strategy for mitigating enteric CH_4_ production from domestic ruminants on forage-based diets. This study assessed a wide variety of dietary CH_4_ mitigation strategies in the laboratory, to provide background for future studies with live animals on the efficiency and feasibility of dietary manipulation strategies to reduce CH_4_ production. Among different chemical and plant-derived inhibitors and potential CH_4_-reducing diets assessed, inclusion of the natural antimethanogenic macroalga *Asparagopsis taxiformis* showed the strongest, and dose-dependent, CH_4_ mitigating effect, with the least impact on rumen fermentation parameters. Thus, applying *Asparagopsis taxiformis* at a low daily dose was the best potential dietary mitigation strategy tested, with promising long-term effects, and should be further studied in diets for lactating dairy cows.

**Abstract:**

We assessed and ranked different dietary strategies for mitigating methane (CH_4_) emissions and other fermentation parameters, using an automated gas system in two in vitro experiments. In experiment 1, a wide range of dietary CH_4_ mitigation strategies was tested. In experiment 2, the two most promising CH_4_ inhibitory compounds from experiment 1 were tested in a dose-response study. In experiment 1, the chemical compounds 2-nitroethanol, nitrate, propynoic acid, p-coumaric acid, bromoform, and *Asparagopsis taxiformis* (AT) decreased predicted in vivo CH_4_ production (1.30, 21.3, 13.9, 24.2, 2.00, and 0.20 mL/g DM, respectively) compared with the control diet (38.7 mL/g DM). The 2-nitroethanol and AT treatments had lower molar proportions of acetate and higher molar proportions of propionate and butyrate compared with the control diet. In experiment 2, predicted in vivo CH_4_ production decreased curvilinearly, molar proportions of acetate decreased, and propionate and butyrate proportions increased curvilinearly with increased levels of AT and 2-nitroethanol. Thus 2-nitroethanol and AT were the most efficient strategies to reduce CH_4_ emissions in vitro, and AT inclusion additionally showed a strong dose-dependent CH_4_ mitigating effect, with the least impact on rumen fermentation parameters.

## 1. Introduction

The global population is growing and, although there is enough food in the world today, there are major differences in how people live. Meat and milk from ruminants are high-quality foods and a large proportion of their production is based on grass, but production is still resource-intensive. Future intensification of agriculture can reinforce negative effects such as greenhouse gas (GHG) emissions, the main contributor to climate change through global warming [1,2].

Methane (CH_4_) is a powerful GHG that plays a key part in global climate change and concentrations have been rising rapidly in the atmosphere over the past decade. Recently published data based on radioactive carbon (C^14^) content in CH_4_ indicate that anthropogenic emissions of CH_4_ in recent decades have been higher than previously estimated [3]. Satellite data [4] suggest that the increased global CH_4_ emissions in the period 2005–2015 were mostly due to increased extraction of shale gas, and that the natural gas and oil industry contributes twice as much CH_4_ emissions as animal agriculture. 

Methanogenesis in the rumen is an essential metabolic process required to remove molecular hydrogen generated during fermentation. The production of CH_4_ is influenced by animal species, age, management, and diet. The Rumen Census project sequenced a wide variety of rumen and camelid foregut microbial communities in many samples from a wide variety of animal species and countries, to identify factors such as diet, host species, or geography causing the greatest variation in CH_4_ emissions [5]. The results showed that rumen archaeal diversity was similar irrespective of host or diet, and a core rumen bacterial population of 67% of the community occurred irrespective of host or diet. The main diversity changes in other bacteria present were caused by diet, and not host genetics [6]. Although dietary strategies for mitigating enteric CH_4_ production in ruminants have been intensively studied, no single most efficient dietary strategy has been identified for dairy cows on forage-based diets.

Methane losses from typical dairy cow diets are 6–7% of gross energy intake, but losses are approximately 3% in feedlot situations, indicating that feeding high-concentrate diets can reduce CH_4_ production [7]. However, recent data indicate that the effect on CH_4_ production of including more grain in dairy cows diets is small [8] and use of this strategy can therefore be questioned. Use of antimethanogenics or plant inhibitory compounds in ruminant diets can also reduce GHG emissions, and has been suggested as an effective and feasible strategy in the livestock sector [9]. Dietary mitigation strategies do not necessarily make food production from ruminants more energy-efficient, but they reduce CH_4_ emissions to the atmosphere and thus immediately slow down global warming [10], contributing to keep the planet within 2 °C of the pre-industrial baseline [11]. The use of CH_4_ inhibitors might be the most immediate and efficient strategy to reduce CH_4_ emissions from dairy cows. The most successful inhibitor suggested in vivo so far is 3-nitroxypropanol that has showed CH_4_ reducing effects when provided to dairy cows in a low dose [12]. The tropical macroalgae *Asparagopsis Taxiformis* is a recent and natural supplement that has shown very promising CH_4_ inhibitory effects in vitro [13]. 

In vitro gas production technique has been developed to evaluate factors influencing digestibility and fermentation kinetics from feeds. The technique has been used to estimate CH_4_ emission with the advantage of screening large number of samples, providing large amount of data points, and allowing accurate predictions of in vivo CH_4_ production [14]. 

This study assessed and ranked a wide variety of dietary CH_4_ mitigation strategies using an automated gas in vitro system, in order to provide background for future in vivo evaluations of dietary manipulation strategies for efficiently reducing CH_4_ production from domestic ruminants. 

## 2. Materials and Methods

Two in vitro experiments were conducted to assess different dietary antimethanogenic compounds. In experiment 1, the dietary CH_4_ mitigating strategies tested comprised six chemical inhibitory compounds at two levels, three plant-derived inhibitory treatments at two levels, five different potentially CH_4_-reducing diets with the active ingredients in two levels except for one of the diets, and two typical grass silage fermentation acids at two levels to mimic different silage fermentation qualities. In experiment 2, the two most promising CH_4_ inhibitory treatments from experiment 1 were tested in a dose-response experiment designed to represent a wide range of treatment levels. 

### 2.1. Experimental Treatments

#### 2.1.1. Experiment 1 

All experimental diets were composed from a control diet that consisted of timothy grass (*Phleum pratense*), rolled barley (*Hordeum vulgare*), and rapeseed (*Brassica napus*) meal in a ratio of 545:363:92 g/kg diet dry matter (DM). The grass and rolled barley originated from Röbäcksdalens research farm in Umeå (63°45’ N, 20°17’ E), Sweden. The rapeseed meal was a commercial solvent-extracted and heat-moisture-treated protein supplement ExPro-00SF (Aarhus Karlshamn AB, Malmö, Sweden). All potential dietary CH_4_ mitigating strategies tested in experiment 1 are listed in Table 1. The chemical compounds 2-nitroethanol (2-NE), propynoic acid, ferulic acid, p-coumaric acid, and bromoform (Sigma-Aldrich Sweden AB, Stockholm, Sweden) were added without replacing any DM of the control diet. Nitrate was added to the control diet to represent one level of 21 g NO_3_/kg DM or 0.0890 g Ca(NO_3_)_2_ × 4H_2_O/g DM (Sigma-Aldrich Sweden AB, Stockholm, Sweden). The nitrate treatment was compared with a zero-nitrate treatment in which 0.0350 g urea/g DM and 0.051 g CaCO_3_/g DM (J.T. Baker BV, Deventer, The Netherland) were added to the control diet to achieve an isonitrogenous and equivalent diet (159 g crude protein (CP)/kg DM). The plant-derived compounds rowan (*Sorbus aucuparia*) berries and the forb fireweed (*Chamerion angustifolium*) were added to replace grass and barley in the control diet, such that the ratio of forage:concentrate was kept constant relative to all other diets. These ingredients were collected in Umeå (63° N, 20° E), Sweden in October and July 2018, respectively. The red seaweed *Asparagopsis taxiformis* (AT) was added in such a small dose in both levels of the treatment that no replacement of control dietary ingredients was made. The seaweed was harvested in the Azores (38.6° N, 28° W), Portugal, in October 2018. Replacements in the potentially CH_4_-reducing diets were also made so that the forage:concentrate ratio was kept constant relative to all other diets and to contain 160 g CP/kg diet DM. Rapeseed oil and oats (*Avena sativa*) were added to replace grass and barley on a DM basis. These ingredients were also collected in Umeå in July 2018. Dried distiller’s grains (Agrodrank 90, Agroetanol, Östergötland, Sweden) replaced rapeseed meal in the control diet and was added to represent an increment of 20 g/kg DM in CP between the levels (CP concentration 160 g/kg DM and 180 g/kg DM, respectively). The CP concentration was made iso-nitrogenous to the control diet for the lowest level when dried distiller’s grain replaced rapeseed meal.

In the treatments where maize (*Zea mays*) silage replaced grass silage, urea was added to make diets isonitrogenous to the control diet. No correction of CP concentration was made in the diet when red clover (*Trifolium pratense*) replaced grass. 

#### 2.1.2. Experiment 2

In experiment 2, AT (0, 0.06, 0.13, 0.25, 0.5, and 1.0 g/kg of diet organic matter (OM)) and 2-NE (0, 0.3, 0.7, 1.3, 2.6, and 5.1 m*M*) were tested in a dose-response experiment comprising six different treatment levels. The same control diet as in experiment 1, of timothy, rolled barley, and rapeseed meal, was used in experiment 2. 

### 2.2. In Vitro Incubations 

The handling of animals in this experiment was approved by the Swedish Ethics Committee on Animal Research (Dnr A 32-16), represented by the Court of Appeal for Northern Norrland in Umeå, and the experiment was carried out in accordance with laws and regulations governing experiments performed with live animals in Sweden. 

Two lactating Swedish Red cows, fed ad libitum on a diet of 600 g/kg grass silage and 400 g/kg concentrate on a DM basis (presenting chemical composition as 509 g/kg of DM, 425 g/kg NDF, and 171 g/kg CP), were used as donor animals of rumen inoculum for all incubations. The rumen fluid from each cow was filtered separately using a double layer of cheesecloth into Thermos flasks that were pre-warmed and flushed with carbon dioxide (CO_2_) prior to collection. Rumen fluid was transported to the laboratory within 15 min. Equal amounts from each cow were immediately blended, strained through four layers of cheesecloth, and added to buffered mineral solution [15] including Peptone^TM^ (pancreatic digested casein; Merck, Darmstadt, Germany) at 39 °C under constant mixing and CO_2_ flushing, to give a buffered rumen fluid solution with a rumen fluid:buffer ratio of 1:4 by volume. 

Prior to each in vitro incubation, dietary ingredients were dried at 60 °C for 48 h and milled in a Retsch SM 2000 cutting mill (Retsch GmbH, Haan, Germany) to pass through a 1-mm screen. Then 1003 ± 38 mg of DM substrate were weighed into serum bottles flushed with CO_2_, and 60 mL of the previously prepared buffered rumen fluid were added. All bottles were placed in a water bath and gently and continuously agitated at 39 °C during an incubation period of 48 h. 

These procedures were repeated for six runs in total and all samples were incubated, with three replicates of each sample. All runs included triplicate bottles with blanks (i.e., bottles with 60 mL of buffered rumen fluid with no sample or treatment in), and samples were randomly allocated to the in vitro incubation bottles and never incubated in the same bottle in more than one run. 

### 2.3. In Vitro Gas Production Measurements and Sampling

Gas production was measured using a fully automated system (Gas Production Recorder, GPR-2, Version 1.0 2015, Wageningen UR), with readings made every 12 min and corrected to the normal air pressure (101.3 kPa) [16].

Measurement of CH_4_ in vitro was performed according to Ramin and Huhtanen [14] on gas samples withdrawn during the incubation period (0.2 mL) from each bottle at 2, 4, 8, 24, 32, and 48 h. Concentration of CH_4_ was determined with a Varian Star 3400 CX gas chromatograph (Varian Analytical Instruments, Walnut Creek, CA, USA) equipped with a thermal conductivity detector. 

Liquid samples of 0.6 mL were collected from the bottles at 8, 24, and 48 h of incubation and immediately stored at −20 °C until analysis of volatile fatty acids (VFA). Liquid samples for ammonia-nitrogen (NH_3_-N) analysis were taken at 8 and 24 h of incubation, and also stored at −20 °C before further analysis. Liquid samples from the replicate treatments between runs were pooled before NH_3_-N and VFA analysis.

After 48 h of incubation, all bottles were removed from the water bath and placed on ice to stop fermentation. The residue was used for in vitro determination of true organic matter digestibility (TOMD). 

### 2.4. Chemical Analysis 

The concentrations of DM and OM in the individual dietary ingredients were quantified by AOAC [17] method 930.15 and method 942.05, respectively. Concentrations of nitrogen were determined by Kjeldahl digestion of 1000 mg sample in 12 M sulfuric acid using Foss Tecator Kjeltabs Cu (Höganäs, Sweden) in a Block Digestion 28 system (SEAL Analytical Ltd., Mequon, WI, USA), followed by determination of total nitrogen by continuous flow analysis using an Auto Analyzer 3 (SEAL Analytical Ltd., Mequon, WI, USA). The samples were analyzed for neutral detergent fiber (NDF) using a heat-stable α-amylase [18] in an ANKOM200 Fiber Analyzer (Ankom Technology Corp., Macedon, NY, USA).

In vitro TOMD was determined for all samples in all runs by analyzing ash-free NDF concentrations in the residues using 07-11/5 Sefar Petex (Sefar AG, Heiden, Switzerland) in situ bags according to Krizsan et al. [19].

Individual VFA concentrations in rumen fluid samples were determined using a Waters Alliance 2795 UPLC system as described by Puhakka et al. [20], and NH_3_-N concentration according to the method provided by SEAL Analytical (Method no. G-102-93 multitest MT7) using AutoAnalyzer 3.

Bromoform concentration in AT was analyzed according to Roque et al. [21] using an Agilent 7890B GC applied to Agilent 7000C triple quad Mass Spectrometer equipped with a ZB-5ms column (Agilent Technologies, Inc. Santa Clara, CA, USA).

### 2.5. Calculations 

Mean blank gas production within run was subtracted from sample gas production. In vivo predicted CH_4_ production was calculated as described by Ramin and Huhtanen [14] as:

CH_4_ = 265 × CH_4_ concentration + total gas production × CH_4_ concentration × 0.55

where total gas production is in mL/g sample, 265 is the total headspace volume (mL), and 0.55 is the ratio of CH_4_ emissions in the outflow gas from the in vitro system. A mean retention time of 50 h (20 h in the first compartment and 30 h in the second compartment), corresponding to the maintenance level of feed intake, was used in model simulations.

Total VFA (TVFA) production was calculated as: the molar proportion of individual VFA were calculated related to TVFA.

TVFA (mmol) = (∑ individual VFA concentration − mean of blank VFA) × 0.06 (amount of buffered rumen fluid)

The molar proportion of individual VFA were calculated related to TVFA.

The in vitro TOMD was calculated as:

$$\mathrm{TOMD}\;\left(g/\mathrm{kg}\right)=\frac{\mathrm{incubated}\;\mathrm{OM}\;(g)-\mathrm{NDF}\;\mathrm{residue}\;\mathrm{corrected}\;\mathrm{for}\;\mathrm{ash}\;\mathrm{and}\;\mathrm{blank}\;(g)}{1000\times \mathrm{incubated}\;\mathrm{OM}\;(g)}$$

### 2.6. Statistical Analysis 

Data on in vivo predicted CH_4_ production and in vitro TOMD from Experiment 1 were analyzed using the MIXED procedure in SAS (SAS Institute Inc., Cary, NC, version 9.4), by a model correcting for random effect of bottle and fixed effect of run and treatment:

Y_ijk_ = µ + T_i_ + R_j_ +B_k_ + e_ijk_

where Y_ijk_ is dependent variable ijk, µ is overall mean, T_i_ is treatment i, R_j_ is run j, B_k_ is bottle k, and e_ijk_ ~ N(0,$\sigma_{e}^{2}$) is the random residual error. Orthogonal contrasts were included for evaluation of control diet vs. treatment and of linear responses to level of treatment.

Data on measured VFA and NH_3_-N concentrations from Experiment 1 were evaluated in a repeated measurements model using the Toeplitz function in the MIXED procedure in SAS (SAS Institute Inc., Cary, NC, USA, version 9.4) (level within treatment was used as subject). The model accounted for effects of treatment and time, and interactions between treatment and time:

y_ij_ = µ + T_i_ + A_j_ +(TA)_ij_ + e_ij_

where y_ij_ is the dependent variable ij, µ is overall mean, T_i_ is treatment i, A_j_ is time j, (TA)_ij_ is interaction between treatment i and time j, and e_ij_ ~ N(0,$\sigma_{e}^{2}$) is the random residual error.

Data on predicted in vivo CH_4_ production, in vitro TOMD, total VFA (TVFA), and molar proportions of individual VFA and NH_3_-N from experiment 2 were subjected to linear and quadratic regression analysis using the REG procedure in SAS (SAS Institute Inc., Cary, NC, USA, version 9.4). Best fit was judged from lowest root mean square error and highest adjusted R^2^.

Effects were considered statistically significant at *p*-value ≤ 0.05.

## 3. Results

The chemical composition of the control diet and the potential CH_4_ reducing diets is shown in Table 2. The AT bromoform concentration was 6.84 mg/g DM.

### 3.1. Experiment 1

Predicted in vivo CH_4_ production derived from analysis of 48 h gas in in vitro incubation of the control diet was 38.7 mL/g DM, in vitro TOMD was 867 g/kg, TVFA was 3.62 mmol, and molar proportion of acetate, butyrate, and propionate was 583, 125, and 237 mmol/mol, respectively. In comparison with the control diet the chemical compounds 2-NE, nitrate, propynoic acid, p-coumaric acid, bromoform, and the plant compound AT, decreased (*p* ≤ 0.01) in vivo CH_4_ predicted production (Table 3). Addition of 2-NE, bromoform, and AT gave the strongest inhibition (*p* < 0.01) of predicted in vivo CH_4_ production among all experimental treatments (97%, 95%, and 99% reduction in the value for the control diet). The reduction in predicted in vivo CH_4_ production achieved by the other compounds ranged between 38% and 64% of the value for the control diet. Surprisingly, none of the potential CH_4_ reducing diets or lactic acid and acetic acid addition affected CH_4_ production in this study (*p* ≥ 0.20). In vitro TOMD was negatively affected by the chemical compounds p-coumaric acid and bromoform (*p* < 0.01), while rapeseed oil inclusion in the diet increased in vitro TOMD compared with the control diet (*p* = 0.04). Propynoic acid and bromoform decreased (*p* ≤ 0.01) TVFA compared with the control diet. Several of the treatments altered the molar proportions of individual VFA. Acetate decreased (*p* ≤ 0.03) on adding 2-NE, propynoic acid, p-coumaric acid, bromoform, AT, or lactic acid to the control diet. For all those treatments except p-coumaric acid and bromoform, there was a concomitant increase (*p* ≤ 0.05) in molar proportions of propionic and butyric acid compared with the control diet. Results of nitrate vs. zero nitrate treatment were: TVFA 2.91 vs. 3.01 mol, acetate 597 vs. 604 mmol/mol propionate 250 vs. 227 mmol/mol and butyrate 87 vs. 123 mmol/mol. 

The molar proportion of isobutyrate, isovalerate and valerate, and NH_3_-N for the control diet and experimental treatments are given in, Table 4. The molar proportions of the branched-chain volatile fatty acids (BCVFA) were altered by many of the CH_4_ mitigating strategies tested. Compared to the control diet, isobutyrate increased (*p* ≤ 0.01) for p-coumaric acid treatment, while for bromoform treatment the molar proportion decreased (*p* ≤ 0.01). The treatments, 2-NE, propynoic, p-coumaric, ferulic acid, AT, lactic acid, and lactic acid + acetic acid, increased (*p* ≤ 0.04), and bromoform decreased (*p* ≤ 0.01) the molar proportion of isovalerate compared to the control diet. Propynoic acid decreased (*p* ≤ 0.05) while bromoform and AT increased (*p* ≤ 0.05) molar proportion of valerate. Results of nitrate vs. zero nitrate treatment were: isobutyrate 6.63 vs. 9.96 mmol/mol, isovalerate 4.01 vs. 4.94 mmol/mol, valerate 16.4 vs. 16.3 mmol/mol, and NH_3_-N concentration 436 vs. 557 mg/L.

Tests for linear effects between the two levels according to Table 1 and the control diet (0 here) revealed no significant effect on in vitro TOMD (*p* = 0.148) for all treatments (data not presented). However, there was a significant linear decrease (*p* < 0.01) in predicted in vivo CH_4_ production for propynoic acid (24 and 0 mL/g DM) and p-coumaric acid (27.1 and 19.8 mL/g DM) when the inclusion level was increased. 

### 3.2. Experiment 2 

**Figure 1:**
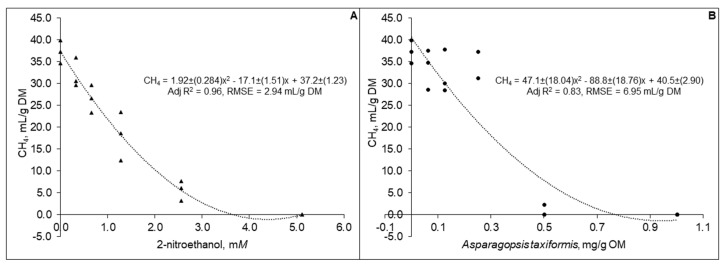
Predicted in vivo methane production based on analysis of 48 h gas from in vitro incubation of a control diet (545:363:92 g/kg of grass silage:barley:rapeseed meal) treated with different levels (three replicates per level) of (**A**) 2-nitroethanol and (**B**) *Asparagopsis taxiformis* in experiment 2.

Predicted in vivo CH_4_ production decreased curvilinearly (*p* < 0.01) with increased levels of both 2-NE (Figure 1). 

**Figure 2:**
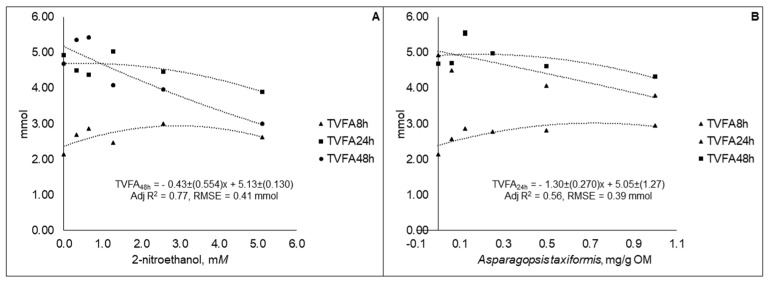
Total volatile fatty acid (TVFA) content in fluid samples taken at different time points during 48 h in vitro incubation of a control diet (545:363:92 g/kg of grass silage:barley:rapeseed meal) treated with different levels (three replicates per level) of (**A**) 2-nitroethanol and (**B**) *Asparagopsis taxiformis* in experiment 2.

The TVFA content decreased linearly (*p* < 0.01) from 5.35 to 3.00 mmol at 48 h for 2-NE and from 4.71 to 4.33 mmol at 24 h for AT for the lower to higher level of supplementation (Figure 2). The TVFA content for 2-NE at 8 h (*p* < 0.01; adj R^2^ = 0.38; RSME = 0.22 mmol) and 24 h (*p* < 0.01; adj R^2^ = 0.55; RSME = 0.25 mmol) showed curvilinear responses, while for AT the curvilinear pattern was verified at 8 h (*p* < 0.01; R^2^ = 0.55; RSME = 0.25 mmol) and 48 h (*p* = 0.01; adj R^2^ = 0.33; RSME = 0.33 mmol).

**Figure 3:**
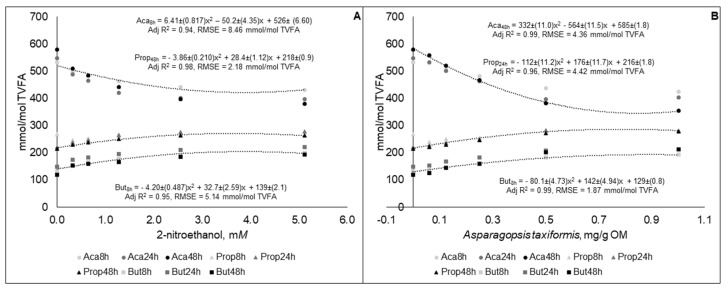
Molar proportions of acetate (Ace), propionate (Prop), and butyrate (But) in fluid samples gas samples taken at different time points during 48 h in vitro incubation of a control diet (545:363:92 g/kg of grass silage:barley:rapeseed meal) treated with different levels (three replicates per level) of (**A**) 2-nitroethanol and (**B**) *Asparagopsis taxiformis* in experiment 2.

Molar proportion of acetate decreased, while propionate and butyrate proportions increased curvilinearly (*p* < 0.01), at all-time points studied for 2-NE and AT (Figure 3). There were no statistical difference (*p* > 0.05) between the coefficients generated for the equations of the different time points. The best fit equations of molar proportions of VFA were generated for both 2-NE and AT from different sampling time points (Figure 3), but the equations generated were not statistically different (*p* > 0.05) from the other sampling time points (results not presented). 

There were no linear or curvilinear relationships between TOMD and level of supplementation for 2-NE (*p* = 0.152) or AT (*p* = 0.142) (results not presented). 

**Figure 4:**
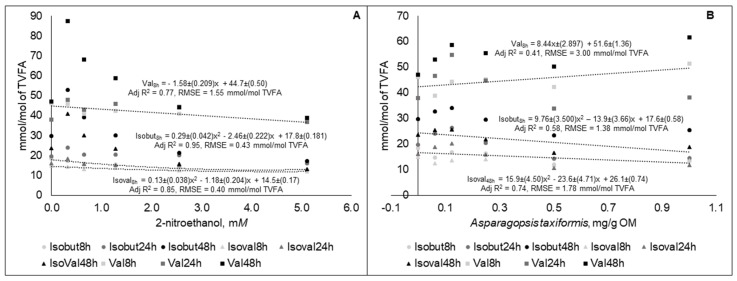
Molar proportions of isobutyrate (Isobut), isovalerate (Isoval), and valerate (Val) in fluid taken at different time points during 48 h in vitro incubation of a control diet (545:363:92 g/kg of grass silage:barley:rapeseed meal) treated with different levels (three replicates per level) of (**A**) 2-nitroethanol and (**B**) *Asparagopsis taxiformis* in experiment 2.

The equations of the molar proportions of BCVFA (isobutyrate, isovalerate, and valerate) were statistically different (*p* < 0.05) between the different sampling time points. The best fit equations are presented in Figure 4.

**Figure 5:**
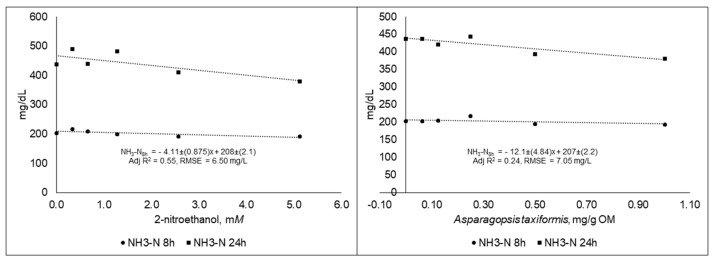
Ammonia concentration (NH_3_-N) in fluid samples taken at different time points during 48 h in vitro incubation of a control diet (545:363:92 g/kg of grass silage:barley:rapeseed meal) treated with different levels (three replicates per level) of (**A**) 2-nitroethanol and (**B**) *Asparagopsis taxiformis* in experiment 2.

The NH_3_-N concentration responses decreased linearly (*p* < 0.05) for both 2-NE and AT at both 8 and 24 h, and the best fit equations are presented in Figure 5.

## 4. Discussion

The CP concentration of the potential CH_4_ reducing diets varied between 140 and 181 g/kg DM, and reflected the characteristics of the dietary ingredient studied in each diets. Regarding that Peptone™ was included in the buffered rumen fluid, none of the diets supplied an insufficient amount of CP in terms of CP available for rumen microbial growth in comparison with in vivo requirements [22]. 

Ruminants are valuable food producers world-wide, since they are able to utilize fibrous non-human-edible resources (forages and pasture) through microbial fermentation of feed in the rumen. Recent data indicate that domesticated ruminants are not the major contributor to anthropogenic CH_4_ emissions. The fermentation of feed and decomposition of manure are the foremost sources of GHG emissions caused by domesticated ruminants [23]. Estimates suggest that livestock are responsible for around 9% and 37% of anthropogenic CO_2_ and CH_4_ emissions, respectively [24]. Long-term strategies to improve feed efficiency through targeted breeding [25] and improved longevity or lifetime productivity [26] could reduce CH_4_ emissions from dairy cows. According to Knapp et al. [9], nutrition and feeding approaches may be able to reduce CH_4_ emissions per unit of energy-corrected milk by 2.5–15%, while reductions of 15–30% can be achieved by combined genetic and management approaches.

### 4.1. In Vitro Measurements of CH_4_ Production in Ruminants

This in vitro study evaluated a wide variety of dietary CH_4_ inhibitors, which would not have been feasible in an in vivo study. A main advantage of the in vitro gas production system in measuring CH_4_ emissions is that it provides a large number of data points, allowing accurate estimates of CH_4_ emissions. However, it is a batch culture approach and has some limitations compared with in vivo studies (e.g., no absorption of VFA over time). The in vitro method used here was developed by Ramin and Huhtanen [14] to overcome this problem and involves a modeling approach based on data obtained from the gas in vitro system. They assumed a gross energy concentration of 18.5 MJ/kg DM, while the predicted proportion of CH_4_ energy for a sample size of around 1000 mg was calculated to be 0.061. This value is close to observed in vivo values at production levels of intake in dairy cows [27]. Recently, Danielsson et al. [28] evaluated the in vitro technique developed by Ramin and Huhtanen [16] using data (diets) from in vivo studies using a respiration chamber to measure CH_4_ emissions. The results showed a high correlation (R^2^ = 0.96) between observed (chamber) data and predicted in vivo CH_4_ values, confirming that the in vitro system is a useful tool for screening diets and evaluating feed additives.

### 4.2. Dietary Strategies to Decrease CH_4_ Production from Ruminants

In this study, we screened many different dietary strategies with known potential to mitigate CH_4_ production from ruminants and also a few new potential inhibitors. It is known that improved forage quality, feeding balanced diets to ensure efficient utilization of nutrients, and optimized microbial protein synthesis in the rumen can decrease CH_4_ production in relation to animal productivity [29]. With respect to improved forage quality, the effects on CH_4_ production reported in the literature are contradictory. Enteric CH_4_ production increases with more digestible substrate available for rumen microbes, but overall emissions of CH_4_ in lactating dairy cows can decrease per kg increase in digestible OM [8]. The mechanism behind this effect is likely that better forage quality improves intake, and thereby increases passage rate. Increased passage rate (i.e., decreased feed retention) and larger animals (i.e., greater body mass) have been associated with reduced CH_4_ emissions in sheep [30,31,32].

Contrasted to our results, in measurements in vivo, rapeseed oil added at 50g/kg DM to a grass silage-based diet reduced ruminal CH_4_ emissions from lactating cows by 22% [33], with the reduction observed being entirely explained by decreases in DM intake and the dilution effect on fermentable OM. Use of dried distiller’s grain to replace soybean meal in diets based on grass silage decreased CH_4_ production in an in vitro study by Franco et al. [34]. The effect was explained by a shift in the ruminal fermentation pattern to decreased acetate and butyrate production and increased propionate production. A similar shift in ruminal fermentation pattern was observed when rapeseed meal replaced soybean meal in vitro in that study [35]. In the present study, dried distiller’s grain replaced rapeseed meal in the control diet and the suggested similarities in ruminal fermentation pattern of these protein supplements would explain the lack of effect on predicted in vivo CH_4_ production. In contrast to our results, Fant et al. [35] observed a significant reduction in predicted in vivo CH_4_ production of 2.1 mL/g DM when using oats instead of barley as the concentrate carbohydrate source. Inclusion of maize silage is also reported to promote propionate fermentation in the rumen, and thereby decrease CH_4_ production in dairy cows [36,37,38]. However, we did not observe this effect with inclusion of maize silage in the diets. Greater molar proportions of acetate and lower proportions of propionate in VFAs when replacing grass with red clover have been reported both in vivo [39] and in vitro [40], suggesting that CH_4_ production potential is greater when ruminants are fed red clover. Maize silage and red clover diets were only numerically lower respectively higher in in vivo predicted CH_4_ production compared with the control diet in this study. On the other hand, grasses are generally more likely to accumulate nitrates than legumes, and nitrate inhibits enteric CH_4_ production by replacing reduction of CO_2_ to CH_4_ as a major sink for disposal of H_2_ in the rumen [41]. Interactions between ruminant physiological responses and diet quality affecting CH_4_ production might explain the lack of impact on in vivo predicted CH_4_ production by the potential CH_4_-reducing diets screened in vitro in this study.

The chemical inhibitors 2-NE and bromoform, and the plant-derived inhibitor AT, gave a very large reduction in predicted in vivo CH_4_ production in this study. The bromoform concentrations of 1.5 and 3.0 mg/kg DM used in this study were representative of concentrations occurring naturally in AT [42]. Vucko et al. [43] analyzed bromoform concentrations in AT biomass subjected to a wide variety of post-harvesting processes and found a maximum concentration of 4.4 mg/g DM for unrinsed, frozen, and freeze-dried AT. Those authors suggest a bromoform threshold of 1.0 mg/g DM in AT for 100% inhibition of CH_4_ production in vitro, which corresponds with our results and the levels used in this study. 

Machado et al. [44] tested different dosages of AT in vitro and found that production of CH_4_ was decreased by 84.7% at an inclusion level of 1% (OM basis), while at AT doses greater than 2% (OM basis), CH_4_ production was decreased by more than 99% compared with the control treatment. In the present study, in vivo predicted CH_4_ production was inhibited almost completely by AT already at a level of 0.5% on an OM basis. Li et al. [45] added AT to diets fed to sheep and observed a reduction of CH_4_ production at inclusion levels exceeding 1% of OM intake, but with altered rumen fermentation at all inclusion rates, i.e., at inclusions ≥0.5% of OM intake. On the other hand, in a short-term in vivo experiment by Stefenoni et al. [46], inclusion of AT at 0.5% of DM intake decreased CH_4_ emission in lactating dairy cows by 80%, with no negative effects on DM intake and milk yield (rumen fermentation parameters were not measured). 

An in vitro study by Zhang and Yang [47] indicated high potential of 2-NE to mitigate CH_4_ production, as also found in the present study. However, they observed a negative effect on in vitro digestibility already at their lowest dose of 5 m*M*, which was not observed in this study. Use of 2-NE in an in vivo trial would not be realistic, considering that the concentration we used in vitro would equate to a daily dose of 0.9 L of 2-NE for a dairy cow with a rumen volume of 200 L.

Except for molar proportion of valerate with increased AT supplementation, all of the BCVFA decreased with increased supplementation in the dose response experiment. The BCVFA are mainly a consequence of the degradation of the amino acids valine, isoleucine, leucine and proline and are used for the biosynthesis of those amino acids and higher branched chain volatile fatty acids. The BCVFA are specific nutrients for the ruminal cellulolytic bacteria, and are believed to have a general positive influence on microbial fermentation [48].

Nitrate, propynoic acid, and p-coumaric acid had much lower inhibitory effects on predicted in vivo CH_4_ production. Nitrate is reported to be an effective CH_4_ production mitigating dietary component [49,50]. For example, a 24.8% reduction in CH_4_ production by lactating cows receiving nitrate at 21.1 g NO_3_^−^/kg of DM was observed by Olijhoek et al. [51]. The dose of nitrate that can be toxic to ruminants’ ranges between 198 and 998 mg/kg live weight and is dependent on diet, administration, and consumption [52]. However, the negative effects of nitrate can be reduced through gradual adaptation of animals to consumption of this nitrogen source, which could contribute to reducing CH_4_ emissions. The CH_4_ inhibitory effect of propynoic acid in this study was lower than that observed by Zhou et al. [53] at a comparable inclusion rate (75.7% reduction compared with a control diet with no inhibitor added). Also the lower amount of TVFA compared with the control diet indicated that propynoic acid can potentially affect digestibility. The inhibitory effect of p-coumaric acid on CH_4_ production by ruminants has not been studied previously and there are no in vitro results with which to compare, but the treatment decreased the dietary TOMD. Lactate in the rumen are metabolized to propionate, which could hypothetical induce changes in ruminal fermentation pattern providing an alternative hydrogen sink to reduce methanogenesis. Likely, the lactic acid preservation has to be more extensive than the levels suggested in this study to have an effect on CH_4_ production in dairy cows.

## 5. Conclusions

This study confirmed that natural bioactives produced by the red seaweed *Asparagopsis taxiformis* can act as a strong natural inhibitor of CH_4_ production in domesticated ruminants. Use of CH_4_ inhibitors with high mitigation potential at a reasonable dietary supplementation level could be an important and effective strategy to mitigate CH_4_ emissions by ruminants. However, *Asparagopsis taxiformis* needs to be further evaluated in vivo to ensure it has no negative effects on animal health, productivity, or product quality. It is also important to establish the long-term CH_4_ mitigation effect of using this inhibitor.

## Figures and Tables

**Table 1:** Experimental treatments evaluated in vitro in experiment 1 for methane (CH_4_) mitigation potential.

Treatments	Levels	
*Chemical compounds*		
2-nitroethanol	5 m*M*	10 m*M*
Nitrate	None ^1^	21 g/kg DM ^2^
Propynoic acid	2 m*M*	4 m*M*
Ferulic acid	10 m*M*	20 m*M*
p-Coumaric acid	10 m*M*	20 m*M*
Bromoform	1.5 mg/g DM	3 mg/g DM
*Plant-derived treatments*		
Rowan berries	50 g/kg DM	100 g/kg DM
Fireweed	50 g/kg DM	100 g/kg DM
*Asparagopsis taxiformis*	10 g/kg OM	20 g/kg OM
*Potentially CH_4_-reducing treatments*		
Rapeseed oil	40 g/kg DM	80 g/kg DM
Dried distiller’s grain	90 g/kg DM	180 g/kg DM
Barley:oat	175:175 g/kg	0:350 g/kg
Maize silage:grass	275:275 g/kg ^3^	545:0 g/kg ^4^
Red clover:grass	275:275 g/kg	None
Lactic acid	60 g/kg DM	120 g/kg DM
Lactic acid + acetic acid	80 + 30 g DM	80 + 60 g DM

DM = dry matter; ^1^ 0.035 g of urea + 0.051 g of CaCO_3_ on DM basis included in control diet in comparison with nitrate treatment; ^2^ 0.089% Ca(NO_3_)_2_ × 4H_2_O on DM basis; ^3^ Urea was added to correct CP at 160 g/kg DM; ^4^ Urea was added to correct CP at 160 g/kg DM.

**Table 2:** Chemical composition (g/kg DM) of control and potential methane (CH_4_) reducing diets evaluated in vitro in experiment 1.

Treatment	Level	Organic Matter	Crude Protein	Neutral Detergent Fiber
Control diet	-----	944	160	387
Rapeseed oil	40 g/kg DM	906	154	372
Rapeseed oil	80 g/kg DM	869	149	356
Dried distiller’s grain	90 g/kg DM	946	161	378
Dried distiller’s grain	180 g/kg DM	946	181	366
Barley: oat	175:175 g/kg	944	165	385
Barley: oat	0:350 g/kg	944	170	383
Maize silage: grass	275:275 g/kg	954	160	355
Maize silage: grass	545:0 g/kg	963	160	323
Red clover: grass	275:275 g/kg	932	171	345
Lactic acid	60 g/kg DM	887	151	364
Lactic acid	120 g/kg DM	831	143	341
Lactic acid + acetic acid	80 + 30 g/kg DM	840	144	345
Lactic acid + acetic acid	80 + 60 g/kg DM	812	140	333

NDF = neutral detergent fibre.

**Table 3:** Effect of experimental treatments on predicted in vivo CH_4_ production (mL/g DM), in vitro true organic matter digestibility (TOMD, g/kg), total volatile fatty acid production (TVFA, mmol), and molar proportions of acetate, propionate, and butyrate (mmol/mol of TVFA) measured in 48 h gas from the in vitro incubation in experiment 1.

Treatment	CH_4_	TOMD	TVFA	Acetate	Propionate	Butyrate	*p*-Value ^1^					
							C_CH4_	C_TOMD_	C_TVFA_	C_Acetate_	C_Propionate_	C_Butyrate_
Control	38.7	867	3.62	583	237	125	–	–	–	–	–	–
2-nitroethanol	1.30	858	3.01	440	309	211	<0.01	0.30	0.10	<0.01	<0.01	<0.01
Nitrate ^2^	21.3	874	2.96	619	250	87	<0.01	0.82	NA	NA	NA	NA
Propynoic acid	13.9	839	2.57	476	297	209	<0.01	0.25	0.01	<0.01	<0.01	<0.01
Ferulic acid	27.5	859	3.54	597	229	109	0.06	0.71	0.82	0.62	0.68	0.32
p-Coumaric acid	24.2	763	3.01	492	176	121	0.01	<0.01	0.10	<0.01	<0.01	0.82
Bromoform	2.00	822	2.30	436	270	261	<0.01	<0.01	<0.01	<0.01	0.08	<0.01
Fireweed	38.1	858	4.01	583	226	140	0.34	0.69	0.24	0.99	0.55	0.34
Rowan berries	28.9	843	3.71	586	241	117	0.96	0.35	0.80	0.93	0.82	0.64
*A. taxiformis*	0.20	852	3.61	418	327	184	<0.01	0.97	0.98	<0.01	<0.01	<0.01
Rapeseed oil	38.2	896	4.04	600	217	128	0.82	0.04	0.24	0.56	0.28	0.83
Dried distiller’s grain	35.3	877	3.74	549	241	152	0.43	0.48	0.73	0.66	0.91	0.33
Barley: oat	37.4	863	3.66	596	225	124	0.73	0.73	0.89	0.65	0.51	0.98
Maize silage: grass	30.7	846	3.71	577	240	136	0.61	0.56	0.80	0.84	0.87	0.48
Red clover: grass	47.9	882	3.16	599	234	129	0.20	0.53	0.27	0.62	0.88	0.85
Lactic acid	34.1	866	3.65	516	271	158	0.25	0.54	0.93	0.03	0.07	0.05
Lactic acid + acetic acid	35.2	885	3.34	598	224	135	0.34	0.11	0.43	0.59	0.49	0.53
SEM	1.75	4.3	0.120	7.2	4.2	5.5	–	–	–	–	–	–

NA = not analyzed; SEM = standard error mean. ^1^ Orthogonal contrasts of control diet vs. treatment of the different in vitro traits. ^2^ Nitrate treatment was compared to the zero nitrate diet made by adding urea and CaCO_3_ to the control diet according to Table 1; numerical differences of TVFA and molar proportions of volatile fatty acids are given in the text.

**Table 4:** Effect of experimental treatments on molar proportions of isobutyrate, isovalerate, and valerate (mmol/mol of TVFA), and ammonia concentration (NH_3_-N, mg/L) measured in 48 h gas from the in vitro incubation in experiment 1.

Treatments	Isobutyrate	Isovalerate	Valerate	NH_3_-N	*p*-Value ^1^			
					C_Isobutyrate_	C_Isovalerate_	C_Valerate_	C_NH3-N_
Control	10.9	0.61	21.4	282	–	–	–	–
2-nitroethanol	5.16	2.77	16.4	436	0.80	<0.01	0.14	0.39
Nitrate ^2^	6.63	4.01	16.43	270	NA	NA	NA	NA
Propynoic acid	8.34	2.59	0.77	311	0.91	<0.01	<0.01	0.25
Ferulic acid	14.4	5.23	20.0	320	0.88	0.03	0.67	0.98
p-Coumaric acid	165	4.23	22.6	263	<0.01	<0.01	0.73	0.81
Bromoform	0.00	0.00	28.7	302	0.64	<0.01	0.03	0.17
Fireweed	10.8	5.97	18.5	304	0.99	0.20	0.38	0.78
Rowan berries	10.1	5.85	21.5	289	0.97	0.15	0.98	0.81
*A. taxiformis*	5.95	5.33	35.4	354	0.83	0.04	0.00	0.52
Rapeseed oil	13.3	8.32	19.1	319	0.92	0.08	0.49	0.25
Dried distiller’s grain	11.5	6.87	20.7	301	0.98	0.91	0.83	0.07
Barley: oat	12.5	6.98	18.5	359	0.95	0.98	0.38	0.75
Maize: grass	10.0	6.10	17.8	281	0.97	0.26	0.28	0.20
Red clover: grass	9.04	5.23	14.6	306	0.94	0.06	0.08	0.45
Lactic acid	9.30	5.29	21.3	323	0.94	0.03	0.97	0.86
Lactic acid + acetic acid	8.95	4.61	16.7	282	0.93	<0.01	0.16	0.77
SEM	4.426	0.370	0.87	12.2	–	–	–	–

NA = not analyzed; SEM = standard error mean. ^1^ Orthogonal contrasts of control diet vs. treatment of the different in vitro traits. ^2^ Nitrate treatment was compared to the zero nitrate diet made by adding urea and CaCO_3_ to the control diet according to Table 1; numerical differences molar proportions of branched-chain volatile fatty acids and NH_3_-N are given in the text.
